# A Diagnostic Model for Alzheimer’s Disease Based on Blood Levels of Autophagy-Related Genes

**DOI:** 10.3389/fnagi.2022.881890

**Published:** 2022-05-12

**Authors:** Qiangqiang Qin, Zhanfeng Gu, Fei Li, Yanbing Pan, TianXiang Zhang, Yang Fang, Lesha Zhang

**Affiliations:** ^1^Second Institute of Clinical Medicine, Anhui Medical University, Hefei, China; ^2^Department of Physiology, School of Basic Medical Sciences, Anhui Medical University, Hefei, China

**Keywords:** Alzheimer’s disease (AD), autophagy, DEGs, nomogram, LASSO

## Abstract

Alzheimer’s disease (AD) is a common neurodegenerative disease. The major problems that exist in the diagnosis of AD include the costly examinations and the high-invasive sampling tissue. Therefore, it would be advantageous to develop blood biomarkers. Because AD’s pathological process is considered tightly related to autophagy; thus, a diagnostic model for AD based on ATGs may have more predictive accuracy than other models. We obtained GSE63060 dataset from the GEO database, ATGs from the HADb and screened 64 differentially expressed autophagy-related genes (DE-ATGs). We then applied them to Gene Ontology (GO) and Kyoto Encyclopedia of Genes and Genomes (KEGG) analyses as well as DisGeNET and PaGenBase enrichment analyses. By using the univariate analysis, least absolute shrinkage and selection operator (LASSO) regression method and the multivariable logistic regression, nine DE-ATGs were identified as biomarkers, which are *ATG16L2*, *BAK1*, *CAPN10*, *CASP1*, *RAB24*, *RGS19*, *RPS6KB1*, *ULK2*, and *WDFY3*. We combined them with sex and age to establish a nomogram model. To evaluate the model’s distinguishability, consistency, and clinical applicability, we applied the receiver operating characteristic (ROC) curve, C-index, calibration curve, and on the validation datasets GSE63061, GSE54536, GSE22255, and GSE151371 from GEO database. The results show that our model demonstrates good prediction performance. This AD diagnosis model may benefit both clinical work and mechanistic research.

## Highlights

- A diagnostic model for Alzheimer’s disease (AD) based on screening autophagy-related genes (ATGs) may have more predictive accuracy than other models.
- Nine candidate genes were identified and combined with sex and age to establish a nomogram model.
- The validation of this model suggested good agreement between predictions and observations.
- This AD diagnostic model was considered helpful for clinical work and provided a new perspective on mechanistic research for AD.

## Introduction

Alzheimer’s disease (AD) is a neurodegenerative disease with the cardinal symptoms of anterograde memory decline and the impairment of learning and cognition (Soria Lopez et al., 2019). The most common type of senile dementia, AD has an insidious onset and progressive development (Dong et al., 2021; Li et al., 2021). According to 2018 statistical data, over 50 million patients have been diagnosed with AD, and this number is projected to reach 80 million by 2030 (Reddy and Oliver, 2019). Undoubtedly, this increase will bring great burdens and serious challenges for patients, their families, and society (Marrone et al., 2020).

Currently, AD diagnosis requires multidimensional methods. Frequently used clinical diagnostic methods include the Mini-Mental State Examination (MMSE), neuroimaging, electroencephalogram (EEG) analysis and laboratory analyses, such as testing for amyloid β-peptide (Aβ) in cerebrospinal fluid (Cassani et al., 2018; Khan et al., 2020; Mintun et al., 2021). Some of these tests are invasive, unconventional, and costly, making them unacceptable for some patients, particularly elderly individuals (Ralbovsky et al., 2019). In addition, because AD cannot be cured currently and the mean survival time after diagnosis is just 4–8 years among those over 65 (2022), diagnosis at an early stage is critical. Therefore, an accurate and easily implemented method is needed to diagnose and prevent AD.

Due to numerous studies have shown that trace amounts of Aβ can be observed in peripheral blood (Blennow and Zetterberg, 2018), blood analysis holds potential for AD diagnosis. Furthermore, blood sampling is minimally invasive, convenient, and easy for patients to accept (Alawode et al., 2021). However, merely depending on the detection of Aβ levels in the blood is inadequate due to its low specificity (Chang et al., 2021). Therefore, seeking other AD-specific blood biomarkers has become the focal point of researchers (Nous et al., 2021).

Autophagy is a dynamic autodigestive recycling of intracellular proteins and senescent organelles under certain physiological or pathologic conditions and is needed for cell survival and health (Parzych and Klionsky, 2014). For instance, an organism utilizes autophagy to adapt to metabolic stress and prevent genetic injury. Autophagy functions in the processes of inflammation, cancer, neurodegenerative diseases, cardiovascular disorders, and aging (Levine and Kroemer, 2019). In mammals, there are three types of autophagy macroautophagy, microautophagy and chaperone autophagy, with macroautophagy the major type used to eliminate extracellular Aβ deposition, which is most implicated in AD (Li et al., 2017; Zamani et al., 2019). Increasing data indicate that in the neural system, lysosomes are involved in degrading misfolded proteins, aggregated proteins, and damaged organelles (Cerri and Blandini, 2019). If the autophagy-lysosome pathway (ALP) is injured, it may result in misfolded Aβ deposition, a critical mechanism in AD development. Another key mechanism is the abnormal accumulation of empty autophagic vesicles (Kerr et al., 2017). Therefore, some researchers suggested that autophagy holds promise for diagnosing and treating AD (Xu et al., 2021). This study aims to establish a diagnostic model based on differentially expressed autophagy-related genes (DE-ATGs) in peripheral blood. With this new model, we expect to improve the accuracy and convenience of AD diagnosis and offer more candidate biomarkers both for clinical work and a new perspective on mechanistic research for AD.

## Materials and Methods

### Microarray Datasets

Gene Expression Omnibus (GEO)^1^ is an open-access database that includes genetic chips and high-throughput sequencing datasets. We chose two datasets, GSE63060 and GSE63061, according to the following criteria: (1) contains samples of both AD patients and healthy persons; (2) samples are all derived from peripheral blood; and (3) the number of samples is no less than 100. The GSE63060 dataset based on the GPL6947 platform was set as the training set, including 145 samples of peripheral blood cells extract from AD patients and 104 samples from healthy persons. The GSE63061 dataset was set as the validation set, which was based on the GPL10558 platform, including 139 blood samples from AD patients and 134 samples from healthy individuals.

### Data Processing and Screening of Differentially Expressed Autophagy-Related Genes

The human autophagy database (HADb)^2^ collects information from papers published in PubMed and other public biological databases and supplies a list of directly or indirectly AD-correlative genes and proteins (Moussay et al., 2011). A total of 222 autophagy-related genes (ATGs) were acquired from the HADb. By using the justRMA function in the limma software package (Ritchie et al., 2015), we normalized the expression profile of the training set GSE63060. By using the ComBat function in the sva software package (Gautier et al., 2004), we removed the batch effect and adjusted the background, acquiring the differentially expressed genes (DEGs) with a false discovery rate (FDR) less than 0.05 (Huang et al., 2009; Wang and Wang, 2020). Finally, we identified the intersection of ATGs and DEGs as DE-ATGs, which were analyzed in further steps.

### Comprehensive Analysis of Differentially Expressed Autophagy-Related Genes

DAVID (Huang et al., 2009)^3^ and Metascape (Zhou et al., 2019)^4^ are two comprehensive databases that play important roles in the annotation and visualization of genes, as well as the enrichment of pathways. The Gene Ontology (GO) function enrichment analysis is composed of three main terms: biological process (BP), cellular component (CC), and molecular function (MF). And the Kyoto Encyclopedia of Genes and Genomes (KEGG) enrichment analysis mainly specializes in the enrichment of pathway analysis. DAVID database was utilized to perform a functional analysis of the DE-ATGs, including GO and KEGG. Moreover, Metascape database was also employed to perform DisGeNET and PaGenBase enrichment analyses. *P*-value < 0.05 was set as the cut-off criteria and the top-ten-counts-terms were selected.

### Establishment and Evaluation of the Prognosis With a Risk Scoring Model Based on Differentially Expressed Autophagy-Related Genes

Considering the influence of age and sex on AD development (Podcasy and Epperson, 2016; Muzammil et al., 2021), we set these factors as research variants. In the R environment (version 3.6.1), the tableone package (0.12.0) was applied to screen out the potential factors associated with the diagnosis of AD, and DE-ATG, whose *p*-value was less than 0.05, was considered statistically significant. Then, we adopted a least absolute shrinkage and selection operator (LASSO) regression analysis with the glmnet package (4.1.1), aiming at simplifying the parameter of our model to avoid overfitting (Zhang et al., 2020). We chose the fittest λ and deleted some genes that partially exhibited collinearity to minimize bias. After a multivariable logistic regression analysis of the LASSO regression-produced influencing factors, we chose the relative parameters whose *p*-values were less than 0.05 as the final parameters of the predictive model. We calculated the risk score through a linear combination of each ATG expression level (α) multiplied by the corresponding coefficient (β). The formula was risk score = α_1_*β_1_ + α_2_ *β_2_ + … + α_*n*_ *β_*n*_. To evaluate the predictive accuracy of this model, we applied the pROC (1.0.11) package using R software to calculate the area under the curve (AUC) of each receiver operating characteristic (ROC) curve.

### Modeling and Validation of a Diagnostic Nomogram

As a simple and easy-to-use two-dimensional image, a nomogram is mainly applied to summarize the specific and statistically significant parameters acquired from a multivariable logistic regression analysis. In the R environment, the rms package (6.1.1) was applied to evaluate the probability of suffering from AD. We summarized all the independent factors analyzed by logistic regression to build a diagnostic nomogram model for AD. To reveal the predictive ability of the risk scoring model based on DE-ATGs, we calculated Harrell’s concordance index, which is the C-index, and drew a calibration curve by using the Hmisc (4.4.2) and rms (6.1.1) packages to compare the differences between predictions and actual observations. Furthermore, to verify the model’s practicality and reliability, we performed the above analyses and decision curve analysis (DCA) on the validation dataset GSE63061. As a new tool, DCA can be applied to the practicality of this model on clinical net benefit under different positive thresholds (Van Calster et al., 2018). Benefit is defined as the gain of AD patients who use the model to diagnose AD and receive corresponding treatment, and the loss is defined as the harm caused by the treatment to a normal individual or patients suffering from other neurological diseases. Additionally, net benefit refers to the disparity between the benefit and loss.

## Results

### Identification of Alzheimer’s Disease-Related Differentially Expressed Genes

**FIGURE 1:**
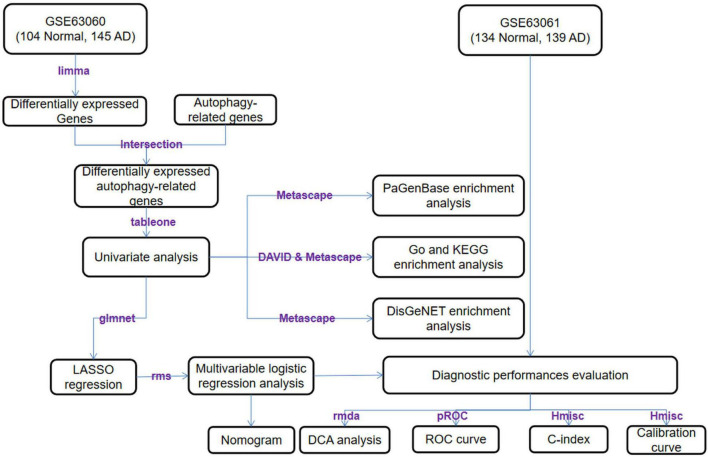
Overview of the workflow.

**FIGURE 2:**
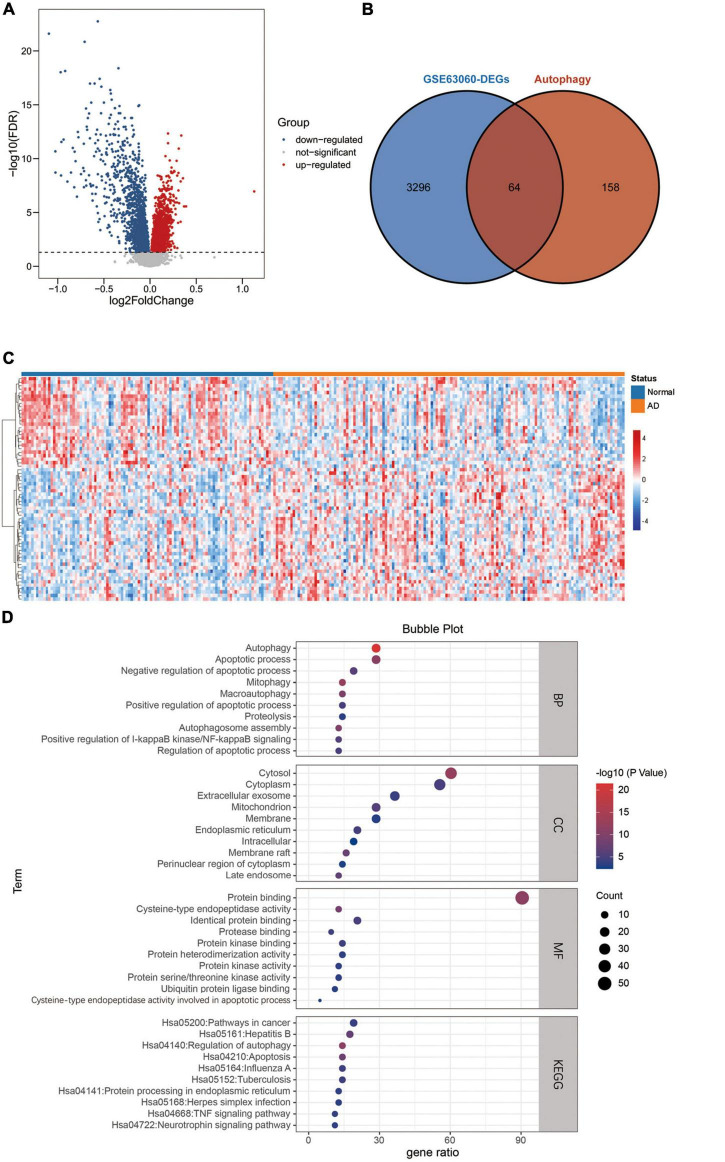
Differentially expressed ATGs. **(A)** The volcano plot for differentially expressed genes in GSE63060. Red represents upregulated genes, blue represents downregulated genes, and gray represents non-significant genes. **(B)** Venn diagram showing the 64 DE-ATGs (the intersection of the DEGs and ATGs). **(C)** A heatmap of DE-ATGs in GSE63060. **(D)** Bubble plot of GO analyses and KEGG pathway analysis of DE-ATGs using DAVID BP: biological process, CC, cellular component; MF, molecular function; KEGG, kyoto encyclopedia of genes and genomes.

An overview of this study is described in the flowchart (Figure 1). Through the analysis of the differentially expressed profiles of 145 cases of AD patients’ blood samples and 104 cases of normal persons’ blood samples, 3360 DEGs with an FDR less than 0.05 were found (Figure 2A). When comparing these DEGs with the 222 ATGs collected from the HADb, 64 identical DE-ATGs were identified (Figure 2B, and their detailed information is shown in Supplementary Table 1). As shown in Figure 2C, the expression levels of the 64 DE-ATGs presented an obvious difference between the AD patients and the normal persons. To discover the potential functional relationship of these DE-ATGs, we utilized DAVID and Metascape to perform a functional analysis of the DE-ATGs, including GO and KEGG. Moreover, we also employed Metascape to perform DisGeNET and PaGenBase enrichment analyses. As the results showed, in addition to autophagy-related pathways, DE-ATGs were generally involved in the BP of apoptotic process, proteolysis, NF-κB signaling pathway. For CC enrichment analysis, DE-ATGs mainly took part in cytosol, cytoplasm, and extracellular exosome. And the identified MF terms were protein binding, cysteine-type endopeptidase activity and protein kinase binding. While the results of KEGG analytical enrichment presented that DE-ATGs took the role in the signaling pathways of pathways in cancer, regulation of autophagy and hepatitis B. The above results were showed in Figure 2D through the gene ratio, count number and *p*-value of genes distributed in different enrichment pathways. The results of genetic functional analysis of DE-ATGs through Metascape were showed in Supplementary Figure 1. All of the above results supported these 64 DE-ATGs for AD discrimination.

### Establishment and Evaluation of the Predictive Risk Scoring Model

**FIGURE 3:**
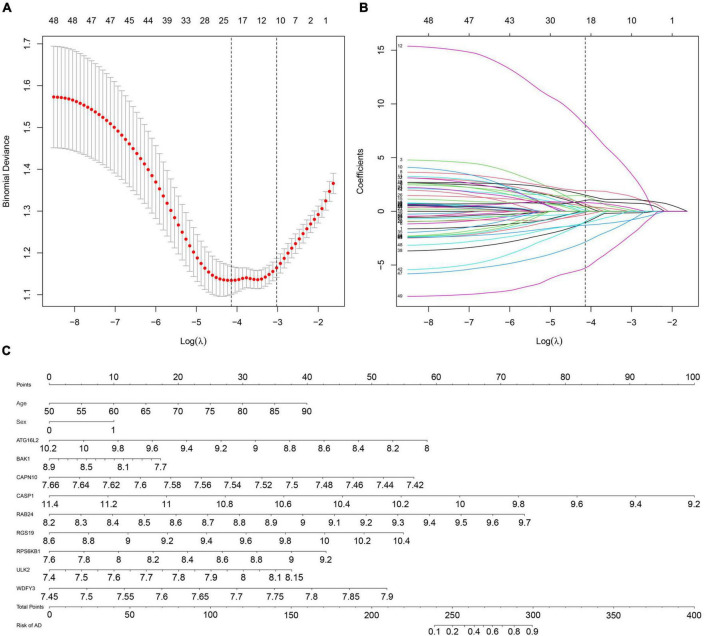
Establishment of a multipredictor nomogram and DE-ATG selection using the LASSO and logistic regression models. **(A)** Cross-validation to select the most suitable tuning parameter lambda (λ); a λ value of 0.014 with log(λ) being –4.233 was chosen as optimal. The first black dotted line represents those 49 features that were reduced to 22 non-zero coefficient features by LASSO. **(B)** The coefficients in the LASSO regression model for key DE-ATGs. **(C)** Predictive nomogram involving age, sex, and the expression profile of 9 DE-ATGs based on selected features.

Next, we used the *T*-test for the normally distributed variables (*T*-value represents the *p*-value) and the Mann–Whitney test for the non-normally distributed variables (*Z*-value represents the *p*-value) to select discriminative gene candidates more effectively. Thus, except for 15 genes with *T*-values or *Z*-values greater than 0.05, 49 of the 64 DE-ATGs were identified for further analysis (see Supplementary Table 1). Considering that a formula containing excessive variables may lead to overfitting and that the genes may exhibit collinearity, we reduced the candidate genes to minimize the bias of this diagnostic model; those genes’ expression levels were shrunk through LASSO regression (see Figure 3A). The most suitable tuning parameter is lambda (λ), which was selected through LASSO regression analysis and cross-validation. In Figure 3A, when log(λ) moves from −8 to −2, the deviance changes accordingly. Then, we found that when log(λ) is −4.233, the minimum deviance can be reached by relying on our model (see the dotted line on the left of Figure 3A). Regarding the beta coefficients (see Figure 3B), which were obtained from LASSO, each curve represents a gene. We made a vertical line when log(λ) equals −4.233 (*x*-axis) and observed the corresponding *y*-value (beta coefficient) of each curve. After removing the genes whose beta coefficient equaled zero when log(λ) equaled −4.233 (*x*-axis), 22 genes remained. Next, we analyzed these 22 genes with logistic regression and found that only nine key DE-ATGs with a *p*-value less than 0.05 were obtained: ATG16L2, BAK1, CAPN10, CASP1, RAB24, RGS19, RPS6KB1, ULK2, and WDFY3. Based on the methods above, we constructed the predictive model using those nine key DE-ATGs. The coefficients of those nine DE-ATGs are listed in Table 1. Thus, to calculate the risk score of each one, the following formula was applied: Risk score = ATG16L2 × (−1.783153) + BAK1 × (−1.248396) + CAPN10 × (−16.275216) + CASP1 × (−3.261934) + RAB24 × (3.608277) + RGS19 × (1.978173) + RPS6KB1 × (1.892426) + ULK2 × (3.958774) + WDFY3 × (7.883500). The nomogram of this model was also built in Figure 3C for visualization and convenient clinical use of the diagnostic model. According to a patient’s actual measured value of nine DE-ATGs’ expression levels in the blood, users could find them on the corresponding scale in the nomogram and project to the point’ scale on the top to read the point of each variant. The sum of every point is the total number of points. The risk probability of this patient suffering from AD could be speculated according to the bottom scale by projecting the total points downward (Figure 3C).

**TABLE 1:** The coefficients and odds ratio (OR) value of 9 DE-ATGs estimated by Logistics regression.

DE-ATGs	Corresponding coefficient (β)	Odds ratio (95% confidence interval)	*p*-value
*ATG16L2*	−2.53	0.08 (0.02,0.38)	0.002
*BAK1*	−2.47	0.08 (0.01,0.93)	0.043
*CAPN10*	−0.48	0.62 (0.42,0.90)	0.013
*CASP1*	−1.39	0.25 (0.12,0.53)	<0.001
*RAB24*	0.78	2.19 (1.21,3.96)	0.010
*RGS19*	1.79	5.97 (1.14,31.35)	0.035
*RPS6KB1*	1.42	4.16 (1.85,9.33)	0.001
*ULK2*	0.62	1.86 (1.24,2.77)	0.002
*WDFY3*	0.51	1.66 (1.03,2.69)	0.038

### Evaluation and Validation of Autophagy-Related Predictive Features

**FIGURE 4:**
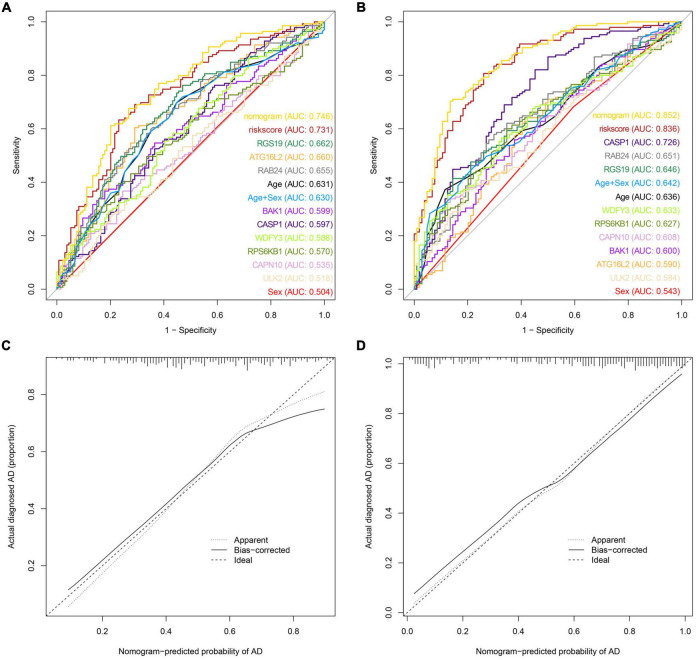
Model discrimination and calibration curve analysis. **(A)** ROC curve for the prognostic model of AD based on GSE63061. **(B)** ROC curve of the AD prognostic model based on GSE63060. **(C)** Calibration curve of the AD nomogram prediction in the GSE63061 set. **(D)** Calibration curve of the AD nomogram prediction in the GSE63060 set.

**FIGURE 5:**
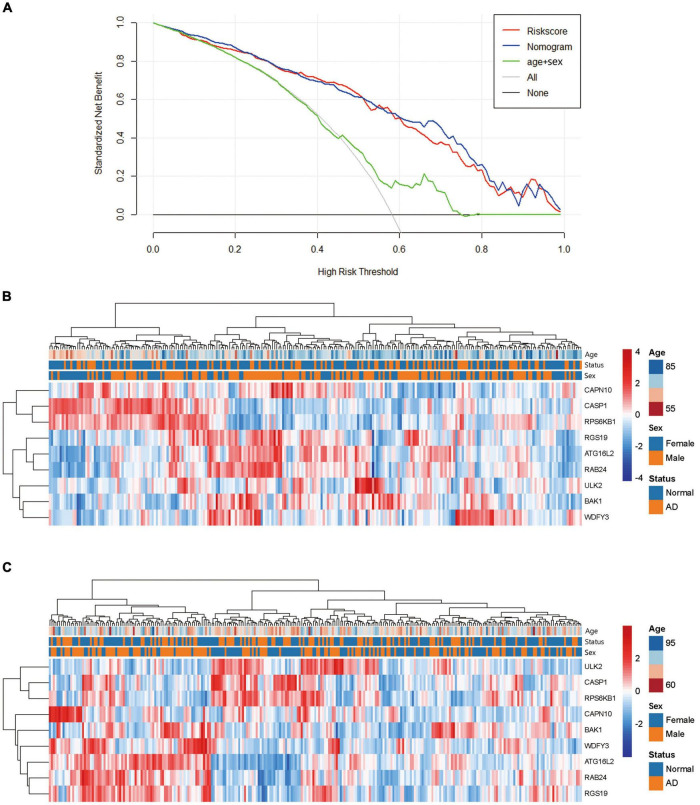
Analysis of the net clinical benefit of the model and the expression patterns of DE-ATGs. **(A)** Decision curve analysis of the predictive nomogram. DCA for the risk score and the model supplemented with clinical parameters. The *y*-axis measures the net benefit. The *x*-axis is the risk threshold probability that changes from 0 to 1. The red line represents the risk score. The blue line represents the nomogram. The green line represents age + sex. The gray line represents the assumption that all patients have thresholds (4–100%), at which using the nomogram to diagnose adds more benefit than the treat-all-patients scheme or the treat-none scheme. **(B)** Heatmap of the DE-ATGs for the prognostic signature in the GSE63060 set. **(C)** Heatmap of the DE-ATGs for the prognostic signature in the GSE63061 set. Expression of the nine selected DE-ATGs in AD patients and normal people. Red represents upregulation. Blue represents downregulation.

We also drew a ROC curve (Figure 4A) to evaluate the predictive accuracy of the AD diagnostic model. The AUC of the ROC curve of the training set GSE63060 was 0.836, demonstrating the model’s good predictive ability. Subsequently, we employed this model to validate the GSE63061 dataset, and the results demonstrated that its AUC was 0.731, which confirmed that the predictive accuracy of this diagnostic model was worth approving (Figure 4B). By using the C-index to test the nomogram, the results showed that the C-index of the training set and validation set were 0.836 and 0.731, respectively, indicating that the model possesses identifying ability. Additionally, the results showed that the AUCs of a model with only the age + sex combination for the two datasets were 0.630 and 0.642, respectively. Notably, these values were less than those from the nomogram of the predictive model. Our predictive model including nine key ATGs improved predictive ability. Furthermore, the calibration curve of the risk nomogram used for predicting the risk of AD showed good consistency between the training set and the validation set (Figures 4C,D). Considering that age and sex are tightly correlated with AD, we calculated the AUC of the nomogram when they were applied to the training set and the validation set. The results were 0.852 for the training set and 0.746 for the validation set, both of which displayed higher accuracy than those without these two factors. As shown in Figure 5A, the DCA curve showed that the threshold of the ratio was 4–100%, indicating that the clinical net benefit was higher when compared with the situation of either no one or all patients using this nomogram for diagnosis. A better net benefit ratio showed a better clinical applicative value. In Figures 5B,C, the heatmap illustrates the expression levels of the nine selected genes *ATG16L2*, *BAK1*, *CAPN10*, *CASP1*, *RAB24*, *RGS19*, *RPS6KB1*, *ULK2*, and *WDFY3* in the two datasets. From these results, compared with the normal control group, *CAPN10*, *CASP1*, and *RPS6KB1* were downregulated in the AD group, while *ATG16L2*, *BAK1*, *RAB24*, *RGS19*, *ULK2*, and *WDFY3* were upregulated in the AD group. Considering that autophagy-related plasmatic factors probably share similar changes among AD, Parkinson’s disease (PD), and stroke, we also tried to analyze GSE54536 (dataset about PD), GSE22255 (dataset about stroke) and GSE151371 (acute CNS injury dataset), respectively, from GEO using R software. The calibration curves of each dataset were shown in Supplementary Figure 2, suggesting that the predicted values of our model are closer to the observed values in the diagnosis of AD than in other diseases and have fine specificity.

## Discussion

Alzheimer’s disease is a common neurodegenerative disease whose specific causes and pathological mechanism have not yet been revealed (Breijyeh and Karaman, 2020). One of the major and key pathological changes is the deposition of Aβ, while the autophagy–lysosome pathway plays a crucial role in the occurrence and development of AD by involving the clearance of Aβ (Li et al., 2017). As age increases, the expression of ATGs reduces, then the oxidative stress aggravates, contributing to the abnormal accumulation of Aβ (Kerr et al., 2017). The evidently enhanced Aβ-induced neural toxicity and delayed dysfunction have a close connection with the downregulation of autophagy activity (Reddy and Oliver, 2019). All the evidence above supports the opinion that increasing age-induced relative autophagy functional disorders are related to the occurrence and development of AD (Omata et al., 2014). Furthermore, different incidence rates have been reported for males and females (Nebel et al., 2018); thus, sex was used as a risk factor in our model. As modern precision medicine develops, biomarkers play an increasingly important role in the treatment and evaluation of therapeutic effects (Hampel et al., 2018). Although the advancement of research on the diagnosis of AD that focuses on Aβ and phosphorylated tau protein can be observed, there are still shortcomings that need to be improved. At present, AD-correlated biomarkers mainly concentrate on molecules in the cerebrospinal fluid (Alawode et al., 2021). However, the sampling process of cerebrospinal fluid is difficult, full of risks and arduous to operate clinically, which is unacceptable for some patients (Ashton et al., 2021). Because of the easy accessibility of blood samples, researches on blood biomarkers for the diagnosis of AD and the discovery of therapeutic targets have broad application prospects (Wang and Wang, 2020; Nous et al., 2021). In this study, we screened DEGs in the blood of AD patients and combined them with autophagy to establish a diagnostic model of AD. We also verified the predictive accuracy and specificity of our model.

In this study, we intersected 3,360 DEGs which were acquired from the datasets in GEO database with 222 ATGs published by the HADb, and then screened 64 DE-ATGs. We screened 49 DE-ATGs that have a close correlation with AD occurrence by the *T*-test and the Mann–Whitney test. Furthermore, we applied LASSO regressive analysis and finally acquired the best regression model containing 22 factors. The logistic regression of the above results revealed nine key DE-ATGs: *ATG16L2*, *BAK1*, *CAPN10*, *CASP1*, *RAB24*, *RGS19*, *RPS6KB1*, *ULK2*, and *WDFY3*. Combined with age and sex, the diagnostic model of AD was established and presented as a nomogram. The AUC value in ROC curve showed good predictive ability. C-index of the nomogram manifested a good distinguishable performance. The calibration curve for the model suggested good agreement between the predictions and actual observations. When applied this model on datasets of PD, stroke, and acute CNS injury, the calibration curve of each dataset (Supplementary Figure 2) suggested fine specificity. This study is the first to combine autophagy with AD-related DEGs to establish a diagnostic model, and reasonably evaluate its predictive accuracy, hopes that it could be applied in the auxiliary diagnosis of AD.

Because the preliminary screening of DEGs from AD patients’ blood is up to 3,360 genes, it cannot indicate the mutable characteristics of AD provided that only single DEG of AD are considered. However, after insertion with ATGs, the range shrunk greatly and showed high specificity. However, this study included a total of 522 sample cases; to some extent, it avoided bias due to limited sample capacity and enhanced credibility. The form of a nomogram could also be more easily accepted and usable by the public.

Compared with normal controls, *ATG16L2*, *BAK1*, *RAB24*, *RGS19*, *ULK2*, and *WDFY3* were upregulated, whereas *CAPN10*, *RPS6KB1*, and *CASP1* were downregulated in AD patients. Studies have shown that starvation could induce the synthesis of ATG16L2 protein in the hepatocarcinoma cell line Huh7 and promote the occurrence of autophagy (Pinto et al., 2016). Patients who suffer from non-small-cell lung cancer (NSCLC) with high ATG16L2 expression have a better prognosis after radiotherapy (Yang and Liu, 2019). Based on several existing mechanistic studies, we speculate that ATG16L2 might strengthen autophagy in patients with AD, while the latter inhibits the abnormal accumulation of Aβ and causes a simultaneous decrease in the proinflammatory factor NOD-like receptor pyrin 3 (NLRP3) (Bai and Zhang, 2021). NLRP3 may induce inflammation of the microglia involved in the genesis of AD (Holbrook et al., 2021). Another apoptosis-related protein, BAK1, is upregulated in AD patients. However, the atypical RAB protein RAB24 is relevant to the transportation of autophagic vacuoles, autophagy–lysosomes, and the clearance of autophagosomes in the late period (Yla-Anttila et al., 2015), and it is involved in ataxia, cancer, etc. (Yla-Anttila and Eskelinen, 2018). Another research suggests that Mir-125b was significantly down-regulated, and the downstream apoptosis-related protein BAK1 was upregulated in a transgenic mouse model of AD (Micheli et al., 2016). *RSG19* is one of the G protein signaling-regulated genes, and the upregulated G protein could negatively regulate G protein-induced signaling transduction by inhibiting the activity of GNAI1, thus resulting in the dysfunction of cholinergic synapses in the nervous system (Xie et al., 2005) and the participation in the development of AD (Silver et al., 2012; Lee et al., 2016). Furthermore, RSG19 could interact with GNAI3 to facilitate the autophagy process (Wu et al., 2014) and modify autophagy by directly detecting extracellular nutrients (Ogier-Denis et al., 1997; Carret-Rebillat et al., 2015; Huang et al., 2020). ULK2 is short for Unc-51-like autophagy activating kinase 2. Among patients with midstage AD, the ULK2 gene is expressed at a relatively high level (Guttula et al., 2012). Ribosomal Protein S6 Kinase (RPS6KB1) showed down-regulated in AD patients, however, when at a low expressed level, it could promote the growth of damaged axons caused by CNS injury (Al-Ali et al., 2017).

The detection method based on the expression of ATGs in peripheral blood to diagnose AD has the advantages of being economical and easily acceptable for targeting individuals with AD prodromal symptoms, and might be converted for clinical application soon. The DE-ATGs candidates could also supply novel targets for treatment and potential mechanism research. However, this study has deficiencies in that ATGs must be updated with the discovery of new genes; thus, there is still room for improvement in this model. The future work also should include collecting more clinical samples to validate the model and further checking the levels of the identified genes in AD’s animal model, such as APPswe/PS1dE9 mouse model (Müller et al., 2021).

In summary, we combined the expression levels of nine DE-ATGs in peripheral blood with age and sex to develop a personalized nomogram model and apply to the diagnostic method of AD. This model could provide a novel insight to medical staff to make preliminarily clinical decisions and supply evidence for future study.

## Data Availability Statement

Publicly available datasets were analyzed in this study. This data can be found here: https://www.ncbi.nlm.nih.gov/geo/query/acc.cgi?acc=GSE63060, https://www.ncbi.nlm.nih.gov/geo/query/acc.cgi?acc=GSE63061, https://www.ncbi.nlm.nih.gov/geo/query/acc.cgi?acc=GSE54536, https://www.ncbi.nlm.nih.gov/geo/query/acc.cgi?acc=GSE22255, and https://www.ncbi.nlm.nih.gov/geo/query/acc.cgi?acc=GSE151371.

## Ethics Statement

This study is not required to obtain ethical approval for it is a secondary analysis based on public datasets. All the datasets analyzed in this study have, respectively, been approved by the corresponding ethics committee. For datasets GSE63060 and GSE63061, ethical approval was received from the Institutional Review Board of the participating institutes; for dataset GSE54536, ethical approval was received from the Ethics Committee of the Research Centre of Neurology, RAMS; for dataset GSE151371, ethical approval was received from the Human Subjects Review Boards at the University of California, San Francisco, and the U.S. Department of Defense Human Research Protection Office; and for dataset GSE22255, ethical approval was received from the ethics committees of the participating institutions. This study does not involve animal experiments.

## Author Contributions

QQ: model design, investigation, methodology, data curation, formal analysis, and writing–original draft. ZG: methodology, data curation, formal analysis, and writing. FL: methodology and writing – original draft. YP: data curation and formal analysis. TZ: investigation and methodology. YF: formal analysis and writing–review and editing. LZ: formal analysis, funding acquisition, and writing – review and editing. All authors contributed to the article and approved the submitted version.

## Conflict of Interest

The authors declare that the research was conducted in the absence of any commercial or financial relationships that could be construed as a potential conflict of interest.

## Publisher’s Note

All claims expressed in this article are solely those of the authors and do not necessarily represent those of their affiliated organizations, or those of the publisher, the editors and the reviewers. Any product that may be evaluated in this article, or claim that may be made by its manufacturer, is not guaranteed or endorsed by the publisher.

## Footnotes

1
http://www.ncbi.nlm.nih.gov/geo/

2
http://www.autophagy.lu/

3
https://david.ncifcrf.gov/

4
https://metascape.org/gp/index.html#/main/step3

## Supplementary Material

Supplementary Figure 1 Genetic functional analysis of DE-ATGs. **(A)** GO and KEGG enrichment analyses of DE-ATGs. **(B)** DisGeNET database enrichment analysis of diseases concerning DE-ATGs. **(C)** Tissue characteristics of DE-ATGs as analyzed using the PaGenBase database.

Supplementary Figure 2 Calibration curve analysis. **(A)** Calibration curve of the PD nomogram prediction. **(B)** Calibration curve of the stroke nomogram prediction. **(C)** Calibration curve of acute CNS injury nomogram prediction. PD = Parkinson’s disease; acute CNS injury = acute central nervous system injury.

## Abbreviations

A β: β -peptide
AD: Alzheimer’s disease
ALP: autophagy-lysosome pathway
ATGs: autophagy-related genes
ATG16L2: autophagy-related protein 16-2
AUC: area under the curve
BAK1: BCL2 Antagonist/Killer 1
BP: biological process
CAPN10: calpain 10
CASP1: caspase-1
CC: cellular component
DCA: decision curve analysis
DE-ATGs: differentially expressed autophagy-related genes
DEGs: differentially expressed genes
EEG: electroencephalogram
FDR: false discover rate
GEO: gene expression omnibus
GO: gene ontology
HADb: human autophagy database
KEGG: kyoto encyclopedia of genes and genomes
LASSO: least absolute shrinkage and selection operator
MF: molecular function
MMSE: mini-mental state examination
NLRP3: NOD-like receptor pyrin 3
PD: Parkinson’s disease
RAB24, RAB24: member RAS oncogene family
RGS19: regulator of G protein signaling
ROC: receiver operating characteristic
RPS6KB1: ribosomal protein S6 kinase B1
ULK2: Unc-51 like autophagy activating kinase 2
WDFY3: WD repeat and FYVE domain containing 3.
