# Supplementary material for: A Diagnostic Model for Alzheimer’s Disease Based on Blood Levels of Autophagy-Related Genes
Source: Front Aging Neurosci. 2022 May 12;14:881890. doi: 10.3389/fnagi.2022.881890 (PMC9133665; doi:10.3389/fnagi.2022.881890)
Supplement: Supplementary file 1 [file Table_1.DOCX]

Supplementary Table 1. The expression levels of 64 DE-ATGs in AD group and normal group.

| DE-ATGs | Expression level of AD  n = 145, Mean ± SD/M (p25, p75) | Expression level of normal  n = 104, Mean ± SD/M (p25, p75) | p value | |
| --- | --- | --- | --- | --- |
| ARSA | 8.28 ± 0.22 | 8.21 ± 0.18 | 0.004 |  |
| ATG16L2 | 9.19 ± 0.38 | 9.07 ± 0.41 | 0.022 |  |
| ATG2A | 8.78 ± 0.32 | 8.77 ± 0.32 | 0.899 |  |
| ATG3 | 8.83 (8.72, 9.01) | 8.84 (8.74, 9.14) | 0.133 |  |
| ATG4A | 7.61 ± 0.07 | 7.64 ± 0.07 | < 0.001 |  |
| ATG4C | 8.05 ± 0.18 | 8.14 ± 0.18 | < 0.001 |  |
| ATG7 | 9.14 ± 0.28 | 9.05 ± 0.23 | 0.003 |  |
| ATG9A | 8.79 (8.66, 8.95) | 8.70 (8.57, 8.88) | 0.004 |  |
| BAK1 | 8.25 ± 0.17 | 8.18 ± 0.17 | 0.007 |  |
| BAX | 8.01 (7.93, 8.08) | 8.08 (7.97, 8.19) | < 0.001 |  |
| BCL2 | 8.14 ± 0.15 | 8.18 ± 0.17 | < 0.001 |  |
| BNIP1 | 7.58 ± 0.07 | 7.62 ± 0.07 | < 0.001 |  |
| BNIP3 | 8.20 ± 0.24 | 8.33 ± 0.21 | < 0.001 |  |
| CAPN10 | 7.51 ± 0.03 | 7.52 ± 0.03 | 0.002 |  |
| CASP1 | 10.09 (9.83, 10.35) | 10.53 (10.07, 10.94) | < 0.001 |  |
| CASP3 | 8.00 ± 0.19 | 8.10 ± 0.18 | < 0.001 |  |
| CASP4 | 8.18 ± 0.14 | 8.24 ± 0.14 | < 0.001 |  |
| CFLAR | 11.86 ± 0.40 | 11.64 ± 0.47 | < 0.001 |  |
| CLN3 | 8.76 ± 0.19 | 8.63 ± 0.22 | < 0.001 |  |
| CTSB | 11.13 ± 0.39 | 10.97 ± 0.35 | 0.001 |  |
| CTSD | 10.33 ± 0.36 | 10.18 ± 0.32 | < 0.001 |  |
| CXCR4 | 9.22 (9.06, 9.36) | 9.19 (9.04, 9.31) | 0.444 |  |
| DAPK2 | 8.29 ± 0.28 | 8.19 ± 0.26 | 0.004 |  |
| DDIT3 | 7.92 (7.81, 8.02) | 7.98 (7.85, 8.11) | < 0.001 |  |
| EEF2 | 12.86 ± 0.24 | 12.76 ± 0.26 | 0.001 |  |
| EIF4G1 | 8.18 ± 0.11 | 8.14 ± 0.11 | 0.008 |  |
| FADD | 9.33 ± 0.21 | 9.28 ± 0.22 | 0.069 |  |
| FAS | 7.57 (7.52, 7.65) | 7.61 (7.55, 7.70) | 0.002 |  |
| FOXO3 | 9.23 (9.11, 9.33) | 9.15 (9.04, 9.31) | 0.026 |  |
| GAA | 8.50 ± 0.25 | 8.45 ± 0.29 | 0.168 |  |
| GABARAPL1 | 9.43 ± 0.26 | 9.54 ± 0.32 | 0.003 |  |
| GAPDH | 11.32 (11.21, 11.46) | 11.26 (11.13, 11.39) | 0.005 |  |
| HGS | 10.62 ± 0.24 | 10.55 ± 0.24 | 0.030 |  |
| HSPA8 | 11.46 ± 0.36 | 11.59 ± 0.36 | 0.005 |  |
| IKBKB | 8.27 ± 0.09 | 8.23 ± 0.09 | < 0.001 |  |
| IKBKE | 8.60 ± 0.23 | 8.51 ± 0.24 | 0.002 |  |
| ITGA3 | 7.61 (7.57, 7.66) | 7.68 (7.62, 7.74) | < 0.001 |  |
| KIAA0226 | 7.85 (7.78, 7.93) | 7.81 (7.74, 7.87) | 0.002 |  |
| LAMP1 | 11.39 ± 0.30 | 11.41 ± 0.31 | 0.726 |  |
| MAPK9 | 7.87 ± 0.09 | 7.91 ± 0.09 | 0.003 |  |
| MTMR14 | 9.02 ± 0.16 | 8.95 ± 0.13 | < 0.001 |  |
| NBR1 | 7.57 (7.52, 7.62) | 7.54 (7.48, 7.60) | 0.002 |  |
| P4HB | 10.67 ± 0.23 | 10.67 ± 0.23 | 0.982 |  |
| PRKCD | 9.69 (9.59, 9.77) | 9.59 (9.47, 9.67) | < 0.001 |  |
| PTEN | 9.68 ± 0.41 | 9.64 ± 0.43 | 0.476 |  |
| RAB11A | 9.53 ± 0.38 | 9.56 ± 0.45 | 0.578 |  |
| RAB24 | 9.13 ± 0.23 | 9.02 ± 0.21 | < 0.001 |  |
| RAB5A | 8.39 ± 0.25 | 8.50 ± 0.26 | 0.001 |  |
| RB1CC1 | 8.54 ± 0.38 | 8.56 ± 0.41 | 0.725 |  |
| RELA | 8.40 ± 0.20 | 8.33 ± 0.17 | 0.005 |  |
| RGS19 | 9.45 ± 0.27 | 9.32 ± 0.21 | < 0.001 |  |
| RPS6KB1 | 8.32 ± 0.33 | 8.45 ± 0.28 | < 0.001 |  |
| SAR1A | 7.91 (7.76, 8.15) | 8.06 (7.82, 8.31) | 0.006 |  |
| SERPINA1 | 10.09 ± 0.27 | 10.00 ± 0.22 | 0.002 |  |
| SPNS1 | 8.43 (8.30, 8.54) | 8.32 (8.21, 8.45) | < 0.001 |  |
| SQSTM1 | 10.56 ± 0.34 | 10.47 ± 0.36 | 0.062 |  |
| TBK1 | 8.80 ± 0.29 | 8.83 ± 0.33 | 0.478 |  |
| TM9SF1 | 8.04 ± 0.10 | 7.99 ± 0.10 | < 0.001 |  |
| TNFSF10 | 10.65 ± 0.51 | 10.68 ± 0.49 | 0.639 |  |
| ULK1 | 9.25 ± 0.18 | 9.20 ± 0.18 | 0.051 |  |
| ULK2 | 7.64 (7.59, 7.73) | 7.62 (7.56, 7.67) | 0.023 |  |
| ULK3 | 7.82 (7.75, 7.91) | 7.77 (7.71, 7.82) | < 0.001 |  |
| VAMP7 | 8.92 ± 0.36 | 8.94 ± 0.40 | 0.616 |  |
| WDFY3 | 7.62 (7.59, 7.68) | 7.60 (7.57, 7.63) | < 0.001 |  |
