# Supplementary figures and images for: A Diagnostic Model for Alzheimer’s Disease Based on Blood Levels of Autophagy-Related Genes
Source: Front Aging Neurosci. 2022 May 12;14:881890. doi: 10.3389/fnagi.2022.881890 (PMC9133665; doi:10.3389/fnagi.2022.881890)

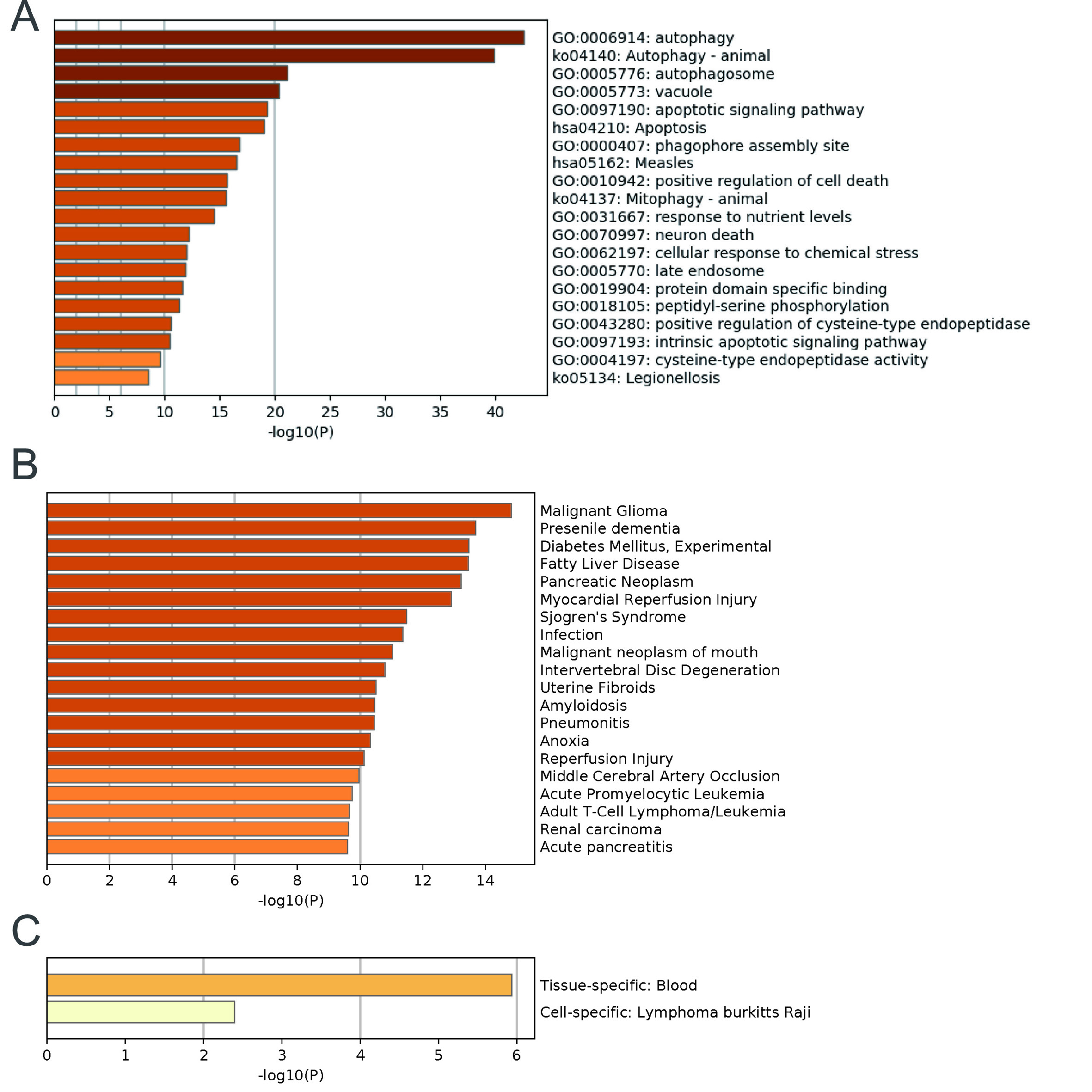

Supplement: Supplementary Figure 1 — Genetic functional analysis of DE-ATGs. (A) GO and KEGG enrichment analyses of DE-ATGs. (B) DisGeNET database enrichment analysis of diseases concerning DE-ATGs. (C) Tissue characteristics of DE-ATGs as analyzed using the PaGenBase database. [file Image_1.JPEG]

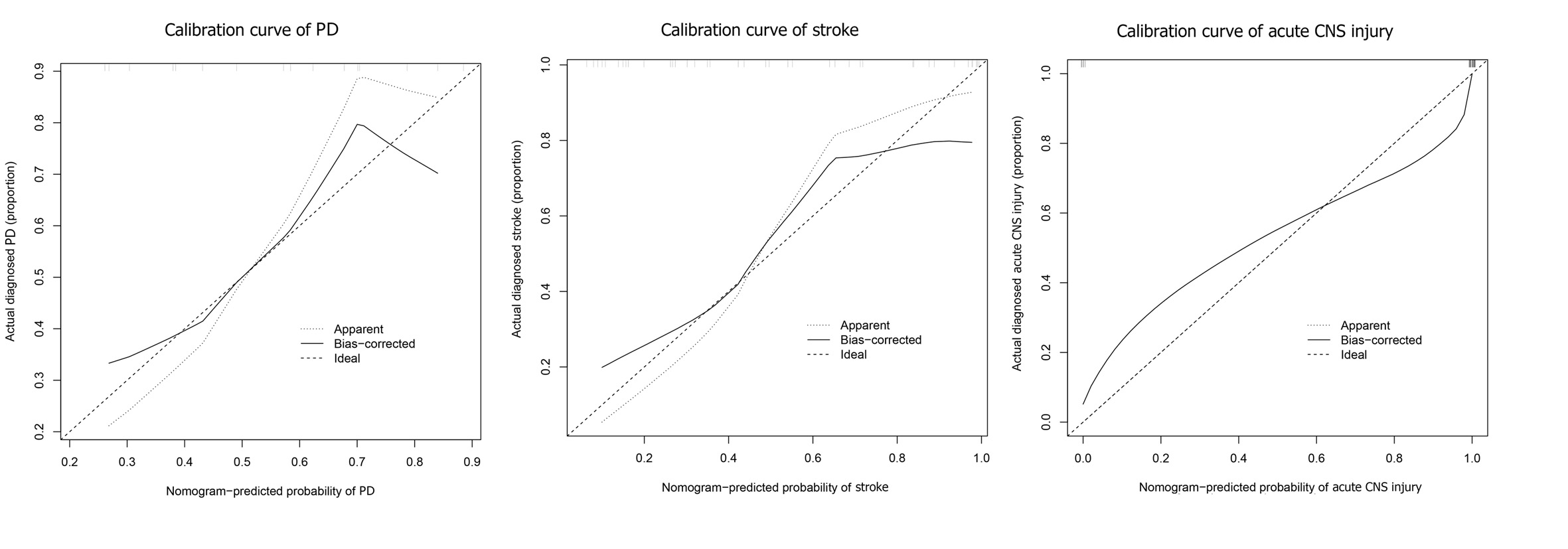

Supplement: Supplementary Figure 2 — Calibration curve analysis. (A) Calibration curve of the PD nomogram prediction. (B) Calibration curve of the stroke nomogram prediction. (C) Calibration curve of acute CNS injury nomogram prediction. PD = Parkinson’s disease; acute CNS injury = acute central nervous system injury. [file Image_2.JPEG]
